# Hematological Profile and Outcomes in Newly Diagnosed Patients with HIV: A 12-Month Single-Centre Cohort Study

**DOI:** 10.3390/v18080831

**Published:** 2026-07-28

**Authors:** Monica-Daniela Padurariu-Covit, Magdalena Miulescu, Pompiliu-Mircea Bogdan, Iulian Stoleriu, Ancuta Elena Tupu, Manuela Arbune

**Affiliations:** 1 Research Centre in the Field of Medical and Pharmaceutical Sciences (ReForm-UDJ), Faculty of Medicine and Pharmacy, “Dunărea de Jos” University of Galati, 800008 Galati, Romania; monica.padurariu@ugal.ro (M.-D.P.-C.); anca.tupu@ugal.ro (A.E.T.); manuela.arbune@ugal.ro (M.A.); 2 Doctoral School of Biomedical Sciences, “Dunărea de Jos” University of Galati, 800008 Galati, Romania; 3 Hematology Department, Emergency County Hospital “Sf. Apostol Andrei”, 800578 Galati, Romania; 4 Anesthesiology and Intensive Care Department, “Sf. Ioan” Emergency Clinical Hospital for Children, 800487 Galati, Romania; 5 Anesthesiology and Intensive Care Department, Emergency County Hospital “Sf. Apostol Andrei”, 800578 Galati, Romania; 6 Faculty of Mathematics, “Alexandru Ioan Cuza” University Iasi, Bvd. Carol I No. 11, 700506 Iasi, Romania; iulian.stoleriu@uaic.ro; 7 Infectious Diseases Clinic Hospital “Sf. Cuv. Parascheva”, 800179 Galati, Romania

**Keywords:** HIV infection, CD4 cell count, viral load, anemia, cytopenias, thrombocytopenia, lymphopenia, survival

## Abstract

Hematological abnormalities are frequent in newly diagnosed people living with HIV (PLWH), yet their prognostic significance remains incompletely defined. We conducted a retrospective single-center cohort study including newly diagnosed PLWH evaluated between 2018 and 2024 and analyzed hematological, immunological, virological, and inflammatory parameters at diagnosis and after 12 months of antiretroviral therapy (ART). Most patients presented with advanced HIV disease and severe immunosuppression. Anemia was the most frequent hematological abnormality and was associated with lower CD4 counts, higher HIV viral load, and reduced survival. Elevated C-reactive protein (CRP) levels and the presence of multiple cytopenias were also associated with poorer outcomes. Notably, patients with two or more cytopenias had significantly reduced overall survival, suggesting that multilineage hematopoietic impairment may reflect advanced systemic disease. Significant hematological and immunological recovery was observed after 12 months of ART. These findings indicate that hematological abnormalities in newly diagnosed HIV infection reflect the interplay between immune dysfunction, systemic inflammation, and impaired hematopoiesis. Multiple cytopenias may serve as readily accessible prognostic biomarkers and could improve early risk stratification in PLWH.

## 1. Introduction

More than four decades after the recognition of the human immunodeficiency virus (HIV) and acquired immunodeficiency syndrome (AIDS) epidemic, advances in modern medicine have enabled effective control of this chronic infection. The widespread availability of effective antiretroviral therapy (ART) and current recommendations advocating rapid treatment initiation following diagnosis have substantially altered the demographic profile of people living with HIV (PLWH), reshaped the epidemiological landscape of HIV infection, and transformed public perceptions of the disease. Nevertheless, the goals of HIV epidemic control and elimination remain unmet in many regions, where persistent challenges include late diagnosis, inequitable access to healthcare services, and delays in treatment initiation [1,2].

In the absence of a curative therapy capable of eradicating the viral reservoir, HIV infection continues to pose a major challenge to contemporary medicine owing to its complex systemic effects, driven by chronic inflammation and persistent immune dysfunction [3]. Hematological abnormalities are among the most common manifestations of HIV infection and include anemia, leukopenia, lymphopenia, and thrombocytopenia, particularly in advanced stages of disease [4]. These abnormalities reflect the complex interplay between viral replication, chronic immune activation, and HIV-induced immunosuppression, resulting in increased peripheral blood cell destruction, dysregulated apoptosis, and impaired bone marrow hematopoiesis [5]. In addition to the direct effects of HIV on hematopoietic progenitor cells and the bone marrow microenvironment, elevated levels of proinflammatory cytokines, including interleukin-1 (IL-1), interleukin-6 (IL-6), tumor necrosis factor alpha (TNF-α), and interferon gamma (IFN-γ), contribute to impaired hematopoiesis and altered differentiation of hematopoietic lineages [6,7]. Persistent systemic inflammation further promotes hepcidin production, disrupting iron homeostasis and contributing to anemia of chronic inflammation [8]. Cytopenias may also be exacerbated by autoimmune mechanisms, opportunistic coinfections, bone marrow infiltration, nutritional deficiencies, and drug-related toxicity [9].

Assessment of hematological parameters together with immunological and virological markers is recommended by current HIV management guidelines as part of the routine evaluation of PLWH. At diagnosis, these investigations are essential for disease staging and assessment of immune status before ART initiation, while subsequent monitoring at 3–12-month intervals is used to evaluate treatment response, immune reconstitution, and disease progression [10,11,12]. Beyond their diagnostic value, hematological abnormalities may provide important prognostic information regarding disease severity and clinical outcomes.

In Romania, contemporary data regarding the hematological profile of newly diagnosed PLWH remain limited. Interest in this topic is driven by the regional epidemiological context, characterized by a persistently high proportion of individuals presenting with late HIV diagnosis. The Romanian HIV epidemic has a distinctive epidemiological profile, initially characterized by a large pediatric cohort associated with probable nosocomial transmission in the early 1990s, followed by a progressive shift toward adult populations, with sexual transmission becoming the predominant route of infection [13]. According to data reported by the Romanian National HIV/AIDS Commission (CNLAS), a cumulative total of 19,315 people living with HIV/AIDS were registered and alive in Romania as of 31 December 2025, while the annual number of newly diagnosed cases has remained relatively stable at approximately 700–800 cases per year [14].

Therefore, this study aimed to evaluate the prevalence of hematological abnormalities present at the time of HIV diagnosis and to investigate their association with survival, virological response, and immunological recovery during the first year of antiretroviral therapy in a Romanian cohort of newly diagnosed PLWH.

Although several international studies have described hematological abnormalities in PLWH, contemporary data from Eastern Europe remain scarce, particularly regarding their prognostic significance in the era of modern antiretroviral therapy. Furthermore, few studies have simultaneously evaluated the relationship between baseline hematological abnormalities, systemic inflammation, virological response, immunological recovery, and short-term survival in newly diagnosed patients. Therefore, the present study aims not only to describe the hematological profile of newly diagnosed PLWH in Romania but also to assess the prognostic value of these readily available biomarkers during the first year after HIV diagnosis.

## 2. Materials and Methods

### 2.1. Study Design and Patient Population

This retrospective, observational, single-center cohort study included 112 newly diagnosed PLWH who were evaluated at the HIV/AIDS Day Care Department of the Clinical Hospital for Infectious Diseases Galați, Romania, between 1 January 2018 and 31 December 2024. Patient evaluation, treatment, and follow-up were performed according to local clinical protocols based on the Romanian versions of the European AIDS Clinical Society (EACS) guidelines [15]. ART was initiated within the first month following confirmation of HIV infection. Study data were collected at two points: at HIV diagnosis and at the completion of the first year of follow-up after diagnosis. Patients who died and those lost to follow-up were identified to determine first-year mortality and retention in care rates following HIV diagnosis.

All participants provided written informed consent for the processing of personal data at the time of hospital admission, in accordance with the General Data Protection Regulation (GDPR). The study was approved by the Institutional Ethics Committee of the Clinical Hospital for Infectious Diseases Galați (Approval No. 3/3/2023). Study data were extracted from the institutional clinical database. Data collection and management were conducted in accordance with the principles of the Declaration of Helsinki, Romanian Law No. 190/2018 on data protection, and the European Union General Data Protection Regulation [16].

#### 2.1.1. Inclusion Criteria

The study included newly diagnosed HIV cases confirmed and reported to the national HIV surveillance database between 1 January 2018 and 31 December 2024, in accordance with the national HIV management guidelines. HIV infection was confirmed by two positive HIV enzyme-linked immunosorbent assay (ELISA) tests and a positive Western blot assay.

#### 2.1.2. Exclusion Criteria

Patients transferred to our clinic after being diagnosed in another HIV treatment center, as well as PLWH diagnosed in other regions of Romania or abroad, were excluded from the study. Cases for which informed consent for the processing of personal data for statistical and research purposes had not been obtained, in accordance with local ethical regulations, were also excluded.

### 2.2. Definitions

The operational definitions used in the study are summarized in Table 1.

### 2.3. Data Collection

**Figure 1:**
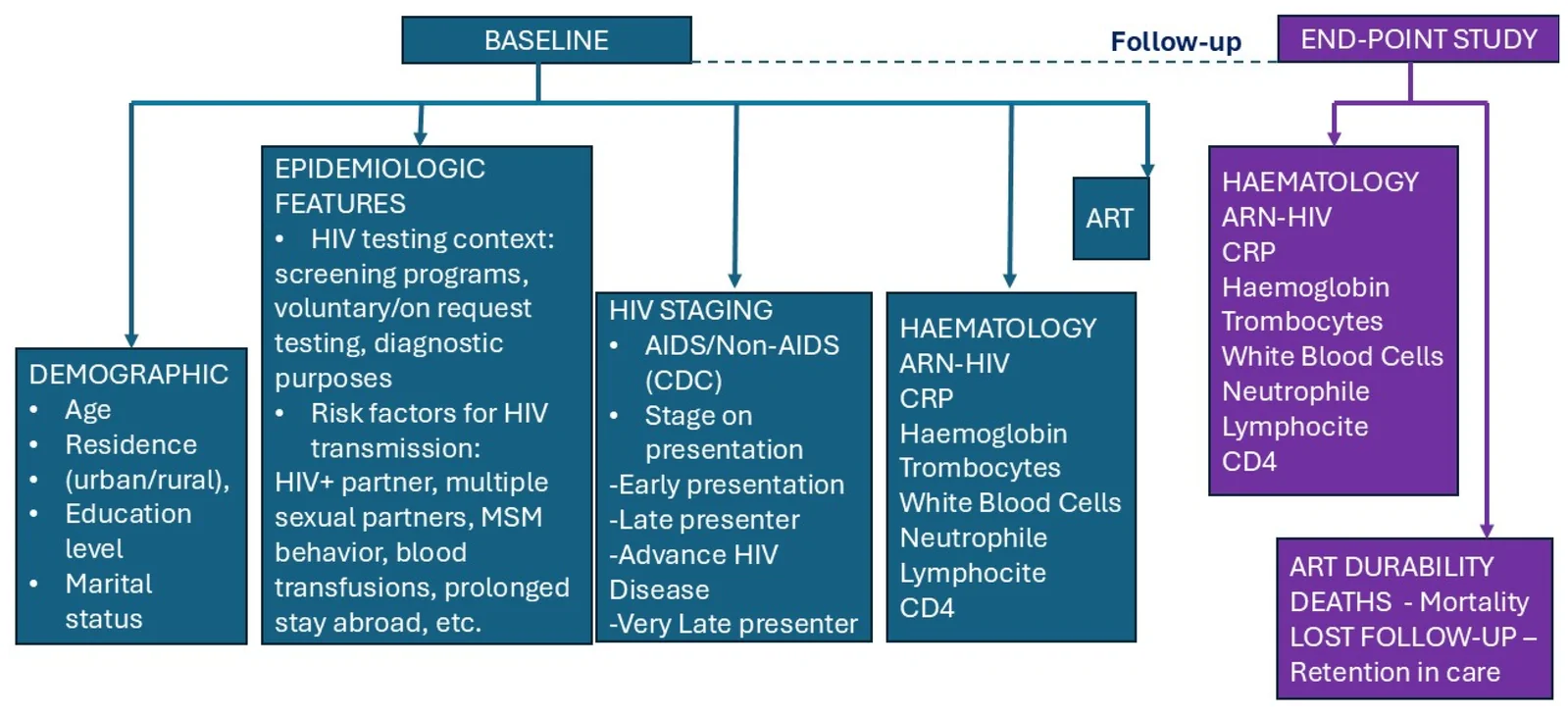
Data collection in the BL and EP.

Demographic and epidemiological data, HIV disease staging information, and hematological, immunological, and virological laboratory parameters were collected at BL (Figure 1).

The context of HIV testing was recorded and categorized as screening programs, voluntary/self-requested testing, or diagnostic testing prompted by clinical suspicion. Epidemiological risk factors for HIV acquisition were also documented, including an HIV-seropositive partner, multiple sexual partners, men who have sex with men (MSM), blood transfusion history, prolonged residence or stay abroad, and other relevant risk exposures.

HIV infection was staged according to the Centers for Disease Control and Prevention (CDC) classification as AIDS or non-AIDS [20,21]. In addition, patients were categorized according to the severity of immunosuppression at diagnosis as early diagnosis, late presenters, advanced HIV disease, or very late presenters, based on the criteria proposed by Antinori et al. [22].

Hematological parameters were assessed longitudinally at BL and EP. Complete blood count analyses were performed using the ABX Pentra XL 80 hematology analyzer (Horiba Medical, Montpellier, France) and included hemoglobin, hematocrit, total leukocyte count, lymphocyte count, neutrophil count, and platelet count. Cytopenias were evaluated both individually (anemia, leukopenia/neutropenia, lymphopenia, and thrombocytopenia) and cumulatively by identifying patients with involvement of one or multiple hematopoietic cell lineages. Hematological parameters were interpreted according to the reference ranges of the local laboratory. Anemia was defined according to the sex-specific lower reference limits of the local clinical laboratory, as hemoglobin (Hb) <12 g/dL in women and <14 g/dL in men, leukopenia as a total leukocyte count <4000/μL, neutropenia as an absolute neutrophil count <2000/μL, lymphopenia as an absolute lymphocyte count <1000/μL, and thrombocytopenia as a platelet count <150,000/μL. CRP levels were measured using the ABX Pentra C400 biochemistry analyzer (Horiba Medical, Montpellier, France) and interpreted according to the laboratory reference values applied throughout the study period [23].

Immunological status was evaluated longitudinally based on CD4+ T-lymphocyte counts, determined by flow cytometry using the CyFlow Counter system (Sysmex Partec GmbH, Görlitz, Germany). Virological status was assessed by plasma HIV RNA levels, measured using the GeneXpert platform (Cepheid, Sunnyvale, CA, USA).

The composition of the initial antiretroviral regimen prescribed by the treating physician was analyzed for all newly diagnosed patients who accepted treatment. ART was initiated in accordance with the current EACS guidelines following screening for opportunistic infections and adherence counseling [12]. At the end of the first year of follow-up, treatment durability was assessed based on the maintenance of the initial ART regimen, while treatment effectiveness was evaluated according to immunological recovery, as reflected by CD4+ T-cell count increase, and virological response, defined as achievement of viral suppression (undetectable HIV RNA).

### 2.4. Statistical Analysis

Statistical analyses were performed using Microsoft Excel 2021 (Microsoft Corp., Redmond, WA, USA) and MATLAB R2024a (MathWorks, Natick, MA, USA).

Statistical analyses included both descriptive and inferential methods. Continuous variables were summarized using means, standard deviations (SDs), 95% confidence intervals (95% CIs), medians, interquartile ranges (IQRs), minimum and maximum values, skewness, and kurtosis, as appropriate. Owing to the highly skewed distribution of HIV RNA levels, viral load values were additionally analyzed after log10 transformation.

Associations between continuous variables were assessed using Spearman’s rank correlation coefficient. Comparisons between patients with HIV RNA levels ≤ 100,000 copies/mL and those with >100,000 copies/mL were performed using the Mann–Whitney U test. Changes in paired hematological parameters between the time of HIV diagnosis and the 12-month follow-up evaluation were analyzed using the Wilcoxon signed-rank test.

To identify independent predictors of the outcome, variables considered clinically relevant and/or associated with the outcome in the univariate analysis (*p* < 0.05) were entered into a multivariable logistic regression model. Adjusted odds ratios (aORs) with 95% CIs were calculated. Statistical significance was defined as a two-sided *p*-value < 0.05. The results of the multivariable analysis were presented as a forest plot showing ORs and corresponding 95% CIs.

## 3. Results

During the first year following diagnosis, four patients were lost to follow-up and seventeen died. Among the 17 deaths recorded during follow-up, the majority were AIDS-related and occurred in patients with advanced HIV disease. The principal AIDS-related causes of death included tuberculosis (*n* = 4), HIV-associated lymphoma (*n* = 3; one probable case without completed diagnostic investigations before death), Kaposi sarcoma (*n* = 2), cerebral toxoplasmosis (*n* = 1), Pneumocystis jirovecii pneumonia (*n* = 1), and progressive multifocal leukoencephalopathy (*n* = 1). Non-AIDS-related deaths were caused by hepatocellular carcinoma (*n* = 1), laryngeal cancer (*n* = 1), ischemic stroke in a patient with previous neurosyphilis (*n* = 1), renal failure (*n* = 1), and one fatal road traffic accident. The retention in care rate at 12 months was 96.4% (108/112), while overall survival at 12 months was 84.3% (91/108).

### 3.1. Demographic and Epidemiological Characteristics

The mean age at HIV diagnosis was 37 ± 12 years (median, 35 years; range, 17–72 years). The cohort was predominantly male (67%), from urban areas (58%), had a low educational level (≤8 years of formal education; 61%), and were single, divorced, or widowed (54.5%).

HIV testing and diagnosis were most frequently performed in symptomatic individuals presenting with clinical manifestations suggestive of immunosuppression (54%; 59/112). Screening programs accounted for 30% (33/112) of diagnoses and included testing during pregnancy, sexually transmitted infections, tuberculosis, viral hepatitis B/C, blood donation, and incarceration. Contact tracing among HIV-positive individuals identified 13% (15/112) of cases, whereas voluntary HIV testing represented only 4% (5/112) of diagnoses.

Evaluation of epidemiological history revealed multiple risk factors for HIV acquisition. The most frequently reported exposures were multiple sexual partners (75.0%), tattoos (65.0%), prolonged residence abroad for more than six months (57.1%), previous surgical interventions (44.6%), dental procedures (44.6%), and having an HIV-positive partner (41.0%). MSM represented 38.0% of the cohort, while 15.2% reported intravenous drug use and 22.5% had a history of incarceration. Previous blood transfusions were reported by 8.0% of patients. Among those with a history of prolonged residence abroad, the majority had lived in the United Kingdom or other European countries.

### 3.2. Baseline Virological and Immunological Profile

The mean baseline HIV RNA level was 494,661 copies/mL. HIV viral load exhibited a markedly right-skewed distribution; however, following logarithmic transformation (log10), the values approximated a normal distribution, with most observations clustered between 5.0 and 5.5 log10 copies/mL (Figure S1). Overall, 58% of patients presented with HIV RNA levels exceeding 100,000 copies/mL at diagnosis.

The mean CD4+ T-cell count at baseline was 267.95 cells/mm^3^ (95% CI: 224.83–311.07). CD4+ T-cell counts also showed a positively skewed distribution and substantial variability, reflecting the heterogeneity of immunological status at the time of diagnosis (Figure S2).

**Figure 2:**
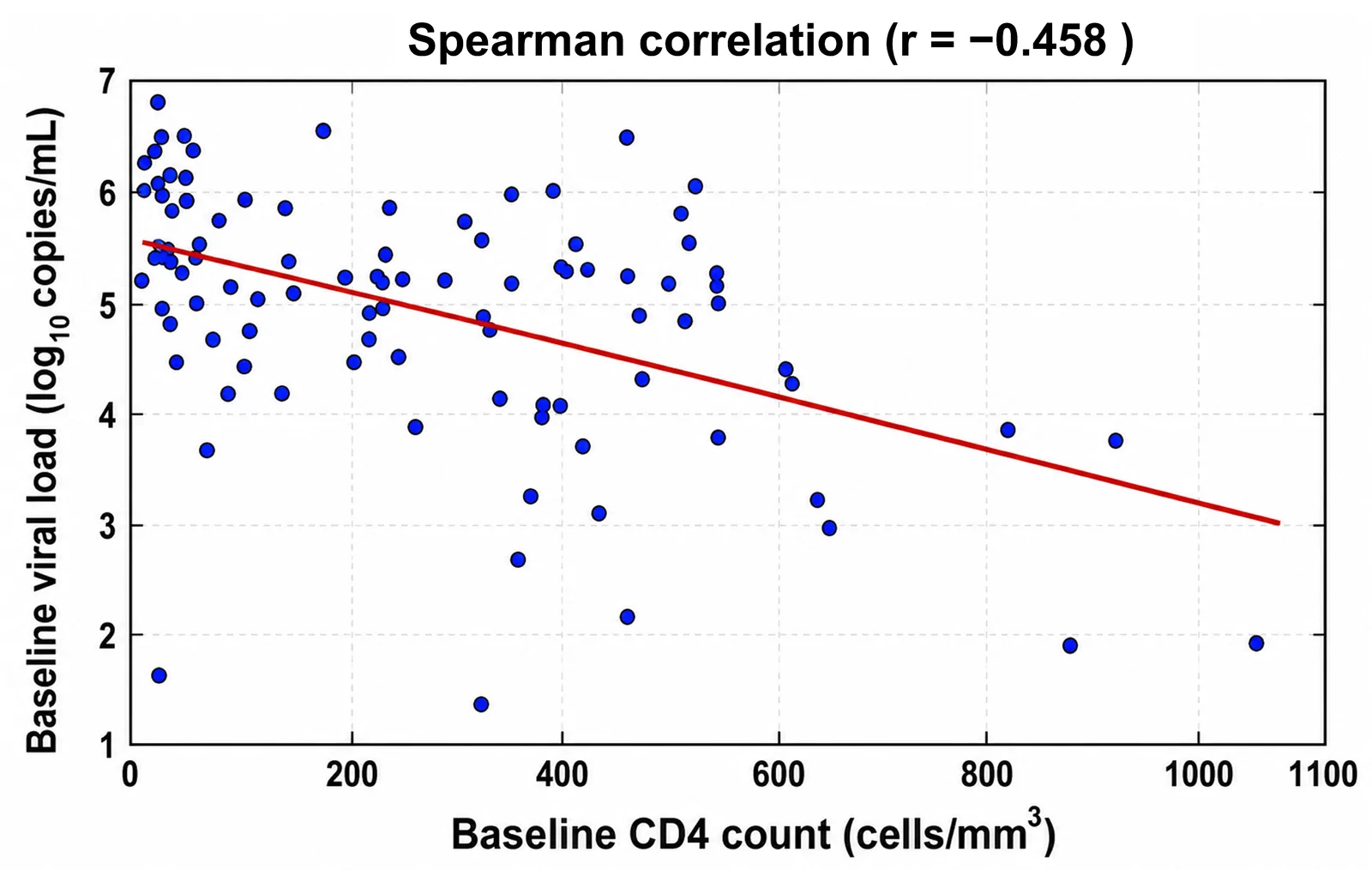
Spearman correlation between CD4+ T-cell count and HIV RNA levels at diagnosis. The red line represents a linear trendline and is shown for visualization purposes only.

A significant inverse correlation was observed between CD4+ T-cell count and HIV RNA level (Spearman’s r = −0.458, *p* < 0.001), indicating that higher levels of viral replication were associated with more profound immunological impairment (Figure 2).

### 3.3. HIV Disease Staging at Diagnosis

According to the revised CDC classification, 49% (55/112) of patients met the criteria for AIDS at the time of diagnosis. AIDS-defining conditions (CDC category C) were present in 41% (46/112) of patients, while 43% (48/112) had a CD4+ T-cell count below 200 cells/mm^3^.

**Figure 3:**
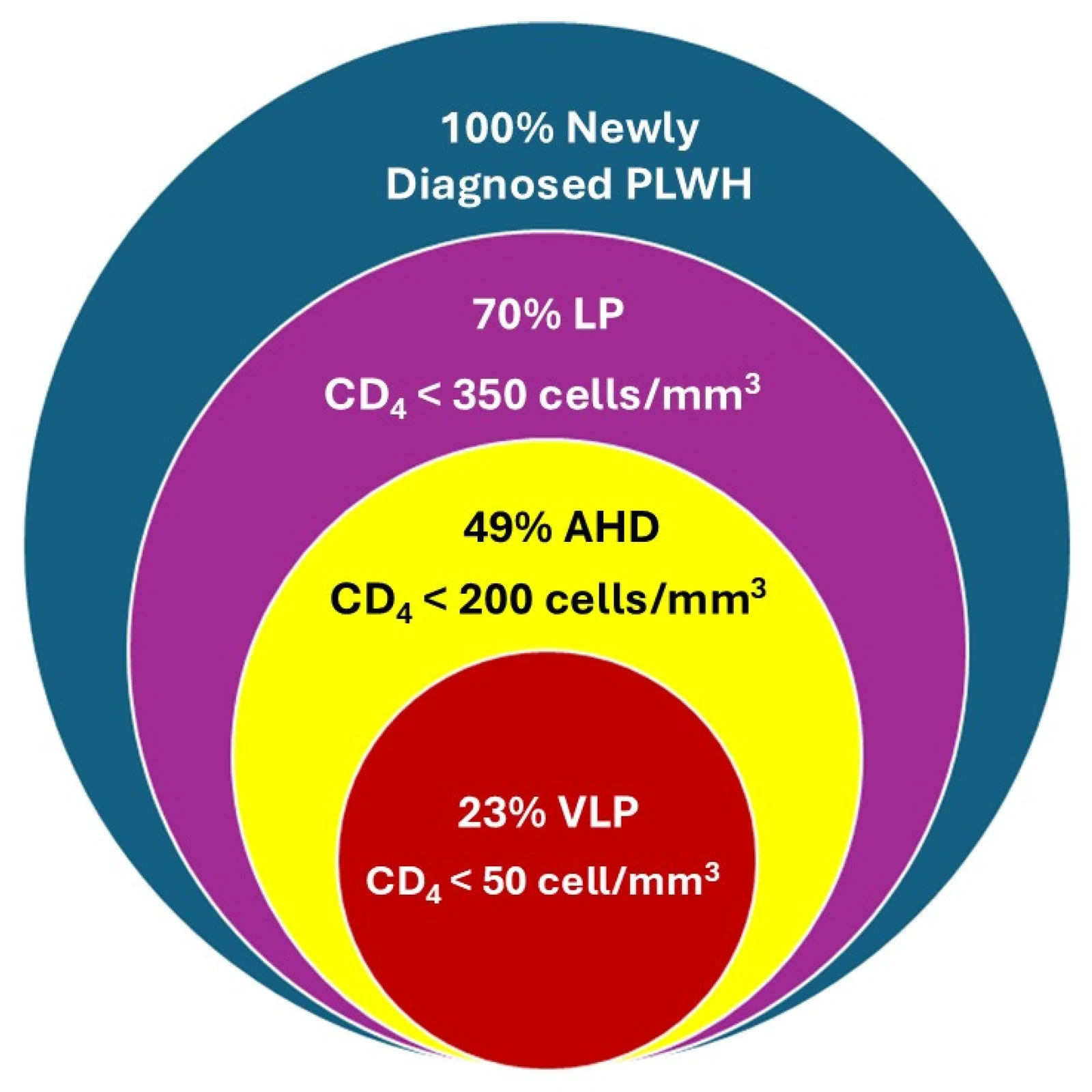
Distribution of newly diagnosed people living with HIV (PLWH) according to disease stage at presentation. Overall, 30% of patients were diagnosed early, whereas 70% fulfilled the criteria for late presentation (LP). Among all newly diagnosed PLWH, 49% had advanced HIV disease (AHD) and 23% were classified as very late presenters (VLP). Abbreviations: LP, late presenter (CD4+ T-cell count <350 cells/mm^3^ or an AIDS-defining condition); AHD, advanced HIV disease (CD4+ T-cell count <200 cells/mm^3^ or an AIDS-defining condition); VLP, very late presenter (CD4+ T-cell count <50 cells/mm^3^).

Based on the consensus definition of late presentation, 70% of patients were classified as late presenters, with CD4+ T-cell counts below 350 cells/mm^3^ at diagnosis. Nearly half of the cohort met the criteria for advanced HIV disease, defined by a CD4+ T-cell count below 200 cells/mm^3^ and/or the presence of an AIDS-defining condition. Furthermore, 23% of patients were classified as very late presenters, with CD4+ T-cell counts below 50 cells/mm^3^ (Figure 3).

### 3.4. First-Line Antiretroviral Therapy

ART was initiated in 90% (102/112) of patients. Individuals who did not receive ART were either diagnosed at a terminal stage of disease and died shortly after diagnosis or were lost to follow-up before treatment initiation.

Initial antiretroviral therapy was predominantly based on integrase strand transfer inhibitors (INSTIs). The most frequently prescribed regimen was bictegravir/emtricitabine/tenofovir alafenamide (33.0%), followed by raltegravir-based regimens (18.7%), elvitegravir/cobicistat/emtricitabine/tenofovir alafenamide (13.2%), dolutegravir/abacavir/lamivudine (9.9%), dolutegravir/lamivudine (8.8%), doravirine/lamivudine/tenofovir disoproxil (7.7%), and efavirenz/emtricitabine/tenofovir (5.5%). The association between the initial ART regimen, virological suppression, and CD4 cell recovery at 12 months is presented in Supplementary Table S1.

Among patients who started ART, the initial treatment regimen remained unchanged during the first year of follow-up in 89% (91/102), demonstrating the durability and overall acceptability of contemporary antiretroviral regimens. Treatment discontinuation was primarily attributable to death during the first year after diagnosis, occurring in the context of advanced immunosuppression and opportunistic diseases.

Most patients (78.5%) received a single-tablet regimen administered once daily, whereas 21.5% were treated with multi-tablet regimens.

Overall, no significant differences in virological suppression or immunological recovery were observed among the different first-line ART regimens. No major ART-related adverse events were reported during the study period.

### 3.5. Baseline Hematological Profile and Virological–Immunological Correlations

Hematological disorders represented the clinical indication for HIV testing in six patients, including one case of severe anemia, one case of severe thrombocytopenia, two cases of non-Hodgkin lymphoma (NHL), one case of Hodgkin lymphoma (HL), and one case of Kaposi sarcoma (KS).

The prevalence of cytopenia at BL varied across the hematopoietic cell lineages evaluated (Table 2).

Although hemoglobin levels were below the lower limit of normal in 34% of patients, most cases of anemia were mild and normocytic in nature. Only two patients (1.7%) presented severe anemia requiring red blood cell transfusion. A strong positive correlation was observed between hemoglobin and hematocrit values (r = 0.967, *p* < 0.001), supporting the internal consistency of the hematological data. Leukopenia was identified in 16% of patients and occurred in association with both lymphopenia (17.9%) and mild neutropenia (26%). Lymphocyte counts showed moderate-to-high variability, with 18.4% of patients presenting absolute lymphocyte counts below 1000 cells/mm^3^. Platelet counts remained within the reference range in most cases, and only one patient required platelet transfusion support due to severe thrombocytopenia (Figure S3A–E).

At HIV diagnosis, 37% of the patients exhibited at least one cytopenia, with 22.7% showing involvement of a single hematologic lineage, 8.2% of two lineages, and 6.2% of all three hematologic lineages. The most common combinations were anemia associated with lymphopenia and anemia associated with thrombocytopenia. The presence of multiple cytopenias suggests more extensive hematological involvement, likely to reflect advanced immunosuppression and persistent immune activation (Table 3).

Baseline HIV RNA levels showed significant negative correlations with hemoglobin concentration (r = −0.299, *p* = 0.002), lymphocyte count (r = −0.349, *p* < 0.001), and platelet count (r = −0.222, *p* = 0.026). In contrast, no significant correlation was observed between HIV RNA levels and neutrophil count (r = 0.029, *p* = 0.852) (Table 4).

CD4+ T-cell count showed a weak but statistically significant positive correlation with hemoglobin concentration (r = 0.374, *p* < 0.001). A moderate positive correlation was observed between CD4+ T-cell count and absolute lymphocyte count (r = 0.584, *p* < 0.001), while a weaker but significant correlation was also identified with platelet count (r = 0.322, *p* = 0.0005). No significant association was found between CD4+ T-cell count and neutrophil count (r = −0.112, *p* = 0.436).

The strongest associations between immunological status and hematological parameters were observed for lymphocyte counts, consistent with the direct cytopathic effects of HIV and the central role of CD4+ T-cell depletion in HIV pathogenesis. In contrast, neutrophil counts appeared to be less closely related to the degree of immunosuppression and may be influenced by additional factors, including intercurrent infections, systemic inflammation, drug-related toxicity, and nonspecific bone marrow dysfunction.

### 3.6. Relationship Between Inflammatory Markers and Hematological Abnormalities

**Figure 4:**
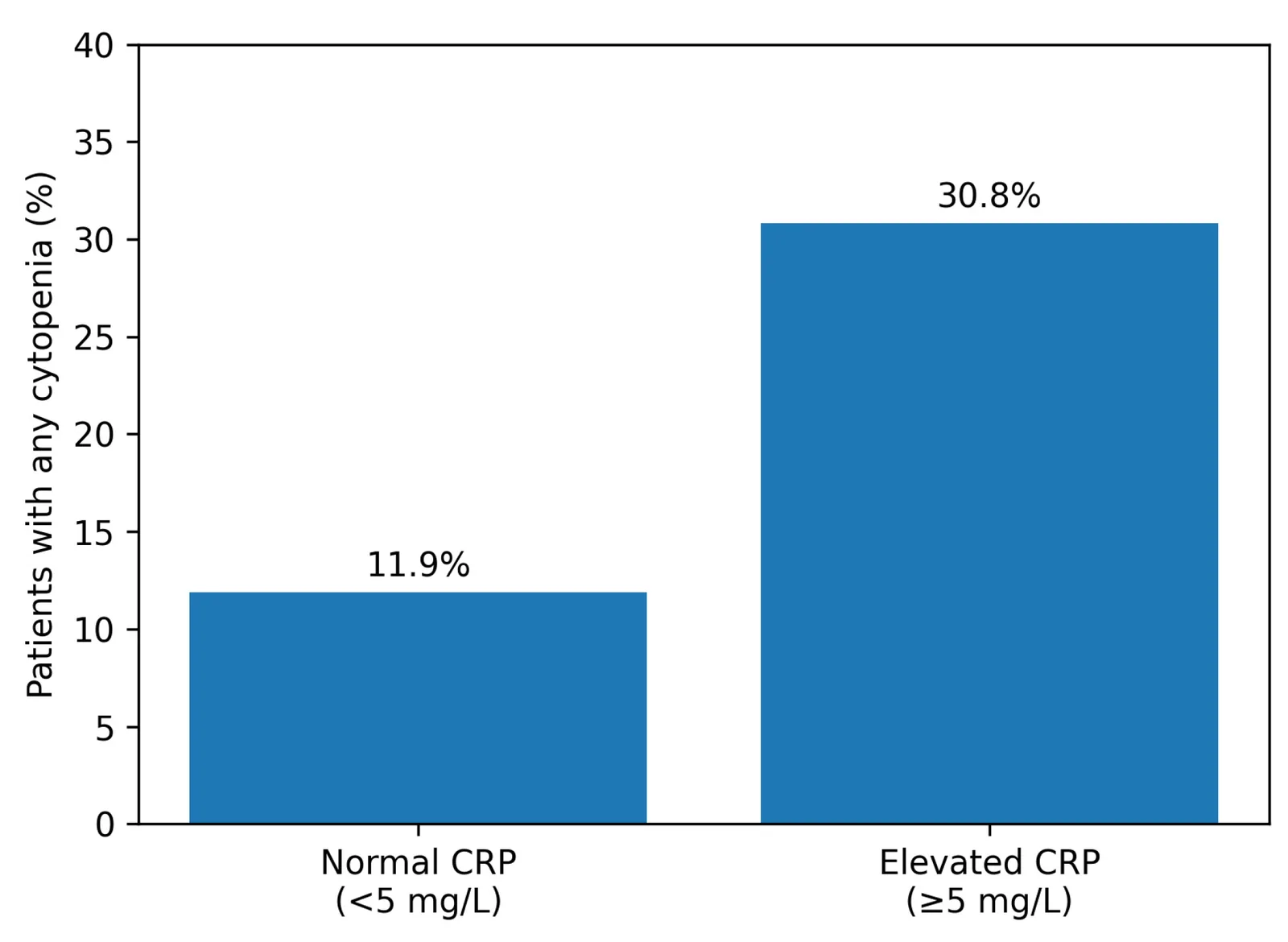
Prevalence of Cytopenia Stratified by CRP Levels.

Among the inflammatory markers evaluated, CRP was the most consistently available parameter. At baseline, the mean CRP level was 11.81 ± 34.61 mg/L, with 28.5% of patients presenting values above the upper reference limit of 5 mg/L. Elevated CRP levels were significantly associated with hematological abnormalities, particularly lower hemoglobin concentrations (Supplementary Table S2). Patients with elevated CRP levels (≥5 mg/L) were more likely to present at least one cytopenia compared with those with normal CRP values 30.8% vs. 11.9%; OR = 3.64, Fisher’s exact test *p* = 0.009 (Figure 4).

### 3.7. Virological, Immunological, and Hematological Status at Follow-Up

At the end of the first year following HIV diagnosis, 81% (74/91) of patients who completed follow-up achieved complete virological suppression, with undetectable HIV RNA levels.

The mean CD4+ T-cell count increased by 212 ± 191 cells/mm^3^ compared with baseline. This increase was highly significant (*p* = 1.1 × 10^−11^), indicating substantial immunological recovery under antiretroviral therapy. Nevertheless, 38% (35/91) of patients continued to have CD4+ T-cell counts below 350 cells/mm^3^, including 34% (31/91) with counts below 200 cells/mm^3^.

No significant differences were observed among antiretroviral treatment regimens with respect to CD4+ T-cell recovery or the achievement of undetectable viral suppression (Table S1).

Longitudinal analysis demonstrated a significant improvement in hematological parameters after 12 months of antiretroviral therapy, accompanied by a marked reduction in the burden of concomitant cytopenias at the individual patient level (Table 5). The proportion of patients without hematological abnormalities increased from 62.9% at baseline to 83.5% at follow-up. Concurrently, the prevalence of multiple cytopenias declined significantly during the first 48 weeks of treatment (*p* < 0.001), reflecting substantial hematological recovery following antiretroviral therapy (Figure S4).

### 3.8. Risk Factors Associated with Mortality

Univariate analysis identified several factors associated with mortality during the first year following HIV diagnosis, including age >40 years, presentation as an advanced late presenter or, particularly, a VLP, anemia, lymphopenia, thrombocytopenia, and elevated inflammatory markers (Table 6).

These findings suggest that individuals older than 40 years presenting with at least one hematological cytopenia and elevated CRP levels may warrant HIV testing, as these readily available clinical and laboratory parameters may serve as indicators of HIV-associated immunosuppression.

**Figure 5:**
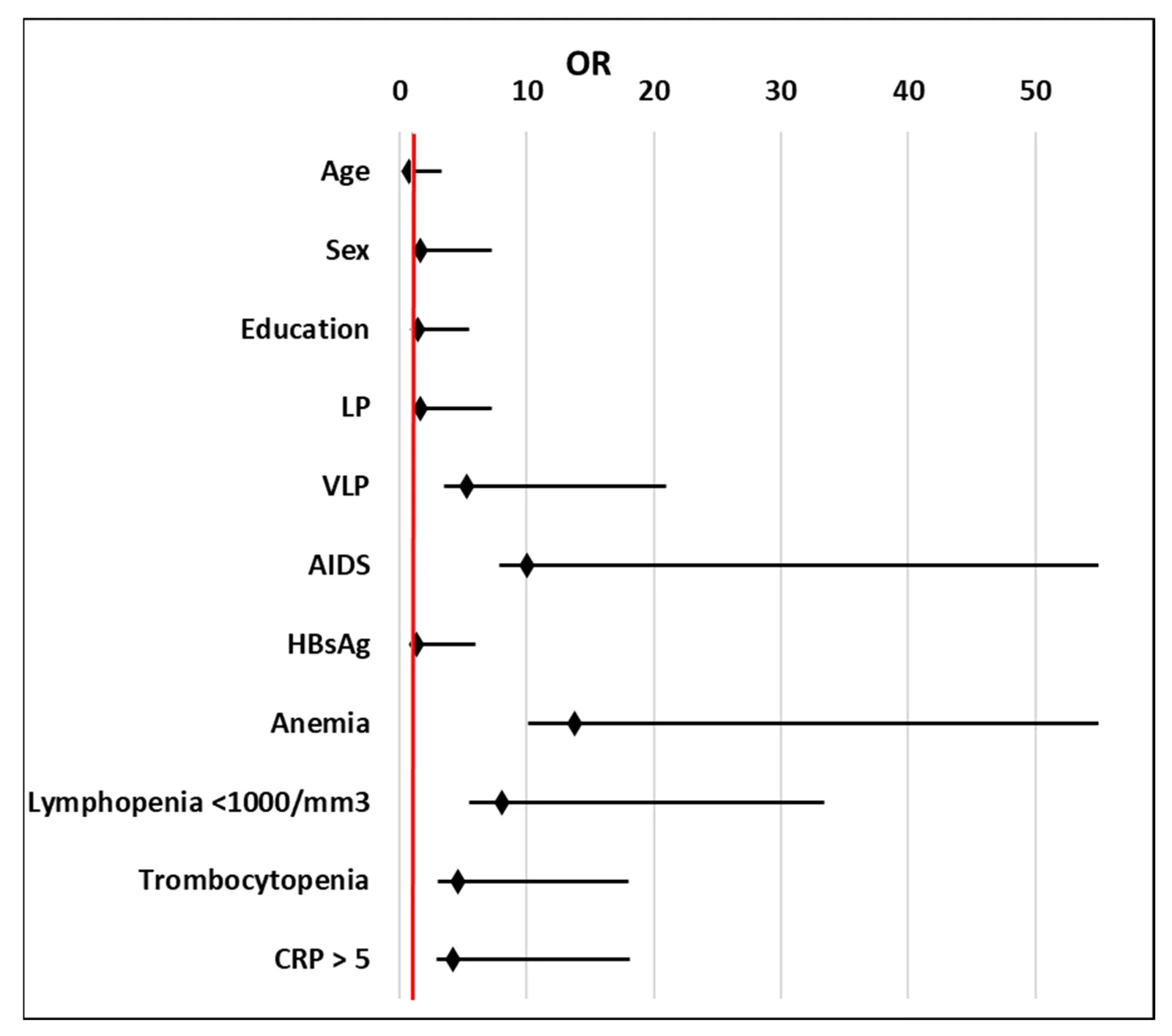
Multivariable logistic regression analysis of factors associated with mortality during the first year after HIV diagnosis. Legend: Forest plot illustrating adjusted odds ratios (aORs) and 95% confidence intervals (CIs) obtained from the multivariable logistic regression model. The red vertical line indicates the null value (OR = 1), representing no association with mortality. Diamonds indicate adjusted effect estimates, and horizontal bars represent 95% CIs. Variables with confidence intervals crossing OR = 1 were not statistically significant. Abbreviations: LP = late presenter; VLP = very late presenter; HBsAg = hepatitis B surface antigen; CRP = C-reactive protein.

In the multivariable logistic regression model, VLP, AIDS, anemia, lymphopenia (<1000/mm^3^), thrombocytopenia, and CRP > 5 mg/dL were included as potential predictors of the outcome. The adjusted odds ratios (aORs) with corresponding 95% confidence intervals (95% CIs) are shown in Figure 5.

In the multivariable logistic regression analysis, several variables emerged as independent predictors of the outcome. VLP, AIDS, anemia, lymphopenia (<1000/mm^3^), thrombocytopenia, and CRP > 5 were significantly associated with increased odds of the outcome after adjustment for confounding factors. These findings suggest that immunological and inflammatory parameters play a central role in determining the outcome, with lymphopenia, thrombocytopenia, anemia, and elevated CRP reflecting systemic immune dysregulation. The strong association observed for AIDS further supports the contribution of baseline immunosuppression to adverse outcomes. The lack of independent associations for demographic variables indicates that biological rather than socio-demographic factors primarily drive the observed risk profile.

## 4. Discussion

The first year following HIV diagnosis represents a critical period in the course of disease, during which patients experience a window of heightened biological vulnerability. Although ART is initiated, immune reconstitution remains gradual, and the risk of opportunistic infections or immune reconstitution inflammatory syndrome (IRIS)-related complications may persist during this period [24,25]. LP has been consistently associated with poorer survival outcomes, delayed immunological recovery, and reduced functional restoration, particularly within the first year after diagnosis [26].

Although 81% of patients who completed follow-up achieved virological suppression after 12 months of ART, a substantial proportion of patients continued to have CD4+ T-cell counts below 350 cells/mm^3^, particularly those presenting with advanced HIV disease. This observation highlights that virological suppression may precede complete immune recovery. Therefore, treatment effectiveness should not be assessed based solely on CD4+ T-cell recovery but should be evaluated by considering both virological suppression and immune reconstitution [2].

Beyond its clinical implications, the first year after HIV diagnosis is characterized by substantial psychological challenges, as patients adapt to the diagnosis, acquire disease-related knowledge, and develop long-term treatment adherence behaviors. Consequently, outcomes observed during the first year following diagnosis may serve as an important indicator of the effectiveness of local healthcare systems in achieving timely HIV diagnosis, rapid treatment initiation, and sustained retention in care [25,27,28].

### 4.1. Late Presentation

Nearly half of all newly diagnosed HIV infections reported in 23 countries of the European Union and European Economic Area (EU/EEA) between 2022 and 2024 were diagnosed at a late stage, substantially exceeding regional targets and highlighting persistent inequalities in access to HIV testing according to age, route of transmission, migrant status, and reporting region within the EU/EEA [28,29].

In a national analysis including individuals newly diagnosed with HIV between 2019 and 2022, approximately 60% of patients were classified as LP and 35% as VLP, confirming that delayed diagnosis remains a major characteristic of the HIV epidemic in Romania [30]. The persistence of a high proportion of late presenters occurs within the context of an HIV epidemic with distinctive molecular characteristics, still predominantly driven by subtype F1 but undergoing progressive diversification through the increasing circulation of subtypes B and A, as well as recombinant forms, suggesting a heterogeneous epidemic landscape [30,31]. Although the direct impact of HIV subtype distribution on late presentation remains unclear, HIV infection continues to be frequently diagnosed at advanced stages, irrespective of recent changes in transmission patterns [32,33].

Our study identified an even higher proportion of late presenters compared with both the EU/EEA average and national estimates, highlighting substantial regional variations and suggesting persistent delays in HIV diagnosis, potentially related to limited access to testing services and insufficient screening among populations at increased risk.

The persistence of a high proportion of late presenters despite the widespread availability of modern antiretroviral therapy suggests the presence of enduring systemic and socio-behavioral barriers, including HIV-related stigma, restricted access to healthcare services, and inadequate testing coverage among vulnerable populations [34]. In this context, the mortality observed in our cohort is comparable to that reported in other European studies, where unfavorable outcomes are predominantly observed among very late presenters and patients with severe immunosuppression at the time of diagnosis [35,36].

The inverse correlation between CD4+ T-cell counts and HIV RNA levels observed in our cohort is consistent with the natural history of untreated HIV infection, reflecting the association between uncontrolled viral replication and progressive immunodeficiency. These findings support the complementary value of HIV RNA and CD4+ T-cell counts as key markers of disease progression at diagnosis [37,38,39].

### 4.2. Clinical Significance of Anemia in HIV Infection

Anemia was one of the most clinically relevant hematological abnormalities identified in our cohort, owing to both its high prevalence and its association with adverse outcomes [5]. The observed relationship between low hemoglobin levels, severe immunosuppression, and elevated HIV viral load supports the multifactorial nature of anemia in HIV infection, involving chronic cytokine-mediated inflammation, impaired bone marrow hematopoiesis, dysregulated iron metabolism, and the systemic consequences of persistent viral replication [39,40,41,42].

In this context, persistent immune activation and chronic inflammation are likely to play a central role in the development of hematopoietic dysfunction through the inhibition of erythropoiesis and disruption of iron homeostasis. The association observed between hemoglobin levels and HIV viral load further supports the link between ongoing viral replication and the severity of hematological impairment.

The clinical relevance of anemia was further supported by the survival analysis, which demonstrated a significantly reduced probability of survival among patients presenting with anemia at the time of HIV diagnosis. These findings are consistent with previous studies identifying anemia not only as the most common hematological manifestation of HIV infection but also as an independent marker of disease progression and mortality [43,44].

From a clinical perspective, the early recognition and appropriate evaluation of anemia may provide valuable information regarding disease severity and help identify patients who require closer monitoring and more intensive clinical follow-up, particularly among those diagnosed at advanced stages of infection.

### 4.3. Lymphopenia and Its Relationship with Virological Activity

Baseline lymphopenia showed the strongest association with HIV viral load, consistent with the well-established tropism of HIV for immune cells and the direct and indirect effects of viral replication on lymphocyte homeostasis [45].

From a biological perspective, lymphopenia reflects not only CD4+ T-cell depletion but also a broader state of immune dysfunction characterized by persistent immune activation, accelerated lymphocyte turnover, and functional exhaustion of cells involved in the antiviral immune response [46]. The observed correlation between CD4+ T-cell counts and total lymphocyte counts further supports the link between HIV-specific immunological damage and global disruption of the lymphoid compartment in advanced HIV infection [9].

In our cohort, the association between lymphopenia and baseline viremia suggests that total lymphocyte counts may indirectly reflect the intensity of viral replication and the degree of immune-inflammatory activation. From a clinical perspective, lymphopenia may represent an easily accessible marker of immunodeficiency severity, particularly in settings where access to advanced immunological assessments is limited.

### 4.4. Thrombocytopenia and Underlying Mechanisms

Thrombocytopenia was more frequently observed among patients with severe immunosuppression and showed a modest inverse correlation with HIV viral load. These findings support the multifactorial nature of HIV-associated thrombocytopenia, involving both immune-mediated peripheral mechanisms and impaired megakaryocytic hematopoiesis within the bone marrow [47].

Proposed mechanisms include immune-mediated platelet destruction through autoantibody production, direct alterations of megakaryocyte function, increased peripheral platelet consumption, and the effects of persistent systemic inflammation on platelet homeostasis. In addition, opportunistic coinfections and certain medications may further contribute to platelet abnormalities in people living with HIV [48].

The relatively modest correlation between thrombocytopenia and HIV viral load suggests that viral replication is not the sole determinant of platelet impairment in HIV infection. Rather, thrombocytopenia appears to reflect a complex interplay between immunosuppression, persistent immune activation, and systemic hematopoietic dysfunction [49].

In recent years, platelets have been increasingly recognized not only as key mediators of hemostasis but also as active participants in the inflammatory and immunological processes associated with HIV infection [5]. From a clinical perspective, the identification of thrombocytopenia may indicate more advanced disease and should prompt careful evaluation for HIV-related complications and comorbid conditions [20,21,33].

### 4.5. Systemic Inflammation and Hematopoietic Dysfunction

Persistent systemic inflammation represents a central feature of HIV infection and contributes not only to the progression of immunodeficiency but also to the development of non-infectious systemic complications [50]. Even in the absence of severe opportunistic infections, chronic immune activation driven by ongoing viral replication may lead to substantial disturbances in hematopoiesis and bone marrow homeostasis [51]. In this context, inflammatory markers may reflect not only the intensity of the immune response but also the extent of systemic involvement associated with HIV infection [52].

The association between elevated CRP levels and lower hemoglobin concentrations supports the hypothesis that inflammatory mechanisms contribute to hematopoietic dysfunction, potentially through activation of pro-inflammatory cytokine pathways and disruption of iron metabolism [9,40]. Increased levels of IL-1, IL-6, TNF-α, and IFN-γ have been shown to inhibit erythropoiesis, promote apoptosis of hematopoietic progenitor cells, and alter iron homeostasis through hepcidin-mediated pathways, thereby contributing to the development of anemia of chronic inflammation. Furthermore, persistent inflammation may impair the bone marrow microenvironment and contribute to multilineage hematopoietic dysfunction [53].

Although the associations between CRP and the remaining hematological parameters did not reach statistical significance, the overall pattern observed in our cohort supports the existence of a complex interplay between immune activation, the severity of immunodeficiency, and HIV-associated hematopoietic dysfunction. These mechanisms may, at least in part, explain the association between multiple cytopenias and the unfavorable outcomes observed in our study population [5,6].

Consistent with these biological mechanisms, patients with elevated CRP levels tended to exhibit poorer survival outcomes, suggesting that persistent systemic inflammation may reflect greater disease activity and more advanced systemic involvement [54]. In contrast, hepatitis B virus (HBV) and hepatitis C virus (HCV) coinfection was not significantly associated with survival in our cohort, although coinfected individuals showed a trend toward less favorable outcomes. These findings differ from those reported in larger cohorts, where viral hepatitis coinfection has been independently associated with increased mortality among PLWH [55]. The discrepancy may be explained by the relatively small number of coinfected patients, the limited follow-up period, and the clinical heterogeneity of the study population.

### 4.6. Hematological Recovery After 12 Months of Antiretroviral Therapy

Longitudinal analysis demonstrated significant improvements in both immunological and hematological parameters following 12 months of antiretroviral therapy. The initial increase in CD4+ T-cell counts after ART initiation is primarily driven by reduced immune activation, which facilitates the redistribution of memory CD4+ T cells from lymphoid tissues into the peripheral circulation. Subsequently, additional mechanisms, including thymic output, peripheral CD4+ T-cell proliferation, and prolonged cellular survival, contribute to the gradual restoration of T-lymphocyte populations during treatment [36].

Although virological suppression remains the primary therapeutic goal of antiretroviral therapy and the principal indicator of treatment efficacy, immune reconstitution follows a more heterogeneous course. In our cohort, 81% of patients who completed 12 months of follow-up achieved virological suppression, representing a favorable virological response despite the high proportion of patients presenting with advanced HIV disease. However, complete CD4+ T-cell recovery was not universal. This finding is consistent with previous studies showing that immune restoration frequently lags behind virological suppression, particularly among patients initiating ART with advanced immunosuppression. Complete CD4+ T-cell recovery may require several years and may remain incomplete in approximately 15–20% of patients who start treatment with CD4+ T-cell counts below 200 cells/mm^3^, despite sustained viral suppression. This discordant viro-immunological response has been associated with irreversible immune damage before ART initiation, persistent immune activation and inflammation, thymic dysfunction, lymphoid tissue fibrosis, older age, and concomitant infections such as cytomegalovirus. Therefore, virological suppression and CD4+ T-cell recovery should be regarded as complementary rather than interchangeable markers of treatment success [56].

The improvement in hemoglobin levels observed in our cohort suggests a reduction in chronic inflammation and in the systemic effects associated with persistent viral replication, resulting in progressive recovery of erythropoiesis [57]. Similarly, the trend toward normalization of platelet counts, particularly among patients with baseline thrombocytopenia, supports the notion that HIV-associated hematopoietic dysfunction is at least partially reversible following effective antiretroviral therapy [58].

These findings confirm that modern antiretroviral therapy exerts benefits that extend beyond virological suppression, contributing to the attenuation of systemic inflammation and the recovery of hematopoietic function. From a clinical perspective, longitudinal monitoring of hematological parameters may provide additional insights into treatment response, the degree of immune reconstitution, and overall systemic recovery among newly diagnosed people living with HIV [41].

### 4.7. Study Relevance and Future Research Directions

Our study provides contemporary data on the hematological profile of newly diagnosed people living with HIV in an Eastern European region where late presentation remains highly prevalent. The findings demonstrate that hematological abnormalities are not merely consequences of immunodeficiency but rather reflect the complex interplay between persistent viral replication, systemic inflammation, and impaired hematopoiesis.

A particularly important finding of our study is the prognostic value of multiple cytopenias. Patients presenting with involvement of two or more hematopoietic cell lineages exhibited significantly reduced survival compared with those without cytopenias or with only a single hematological abnormality [59]. Multiple cytopenias may represent a marker of more advanced systemic disease, characterized by profound immunosuppression, persistent immune activation, and underlying bone marrow dysfunction [60].

Complete blood count parameters, interpreted alongside immunological, virological, and inflammatory markers, represent readily available tools for the early identification of patients at increased risk of adverse outcomes. The integration of cytopenias and inflammatory markers into biological risk models, together with the degree of CD4+ T-cell depletion, may improve prognostic stratification and support the optimization of monitoring and therapeutic strategies for newly diagnosed patients [61].

Our findings also highlight the importance of considering HIV testing in patients presenting with unexplained hematological cytopenia. Anemia, lymphopenia, thrombocytopenia, and other hematological abnormalities may represent early or underrecognized manifestations of HIV infection and should prompt further diagnostic evaluation [62,63]. Incorporating HIV screening into the routine assessment of patients with unexplained cytopenias may facilitate earlier diagnosis, timely initiation of antiretroviral therapy, and ultimately improved clinical outcomes. From both clinical and public health perspectives, optimizing early testing strategies may promote rapid access to ART, sustained viral suppression, prevention of progressive immunodeficiency, and reduce HIV transmission [57,64].

Although our findings suggest that the presence of multiple cytopenias may represent a useful prognostic marker in newly diagnosed PLWH, these observations should be validated in larger prospective multicentre studies before being incorporated into routine clinical risk stratification.

The clinical spectrum of HIV infection has changed substantially since the introduction of effective antiretroviral therapy, hematological abnormalities remain common and continue to pose important diagnostic and therapeutic challenges. Increased awareness of these manifestations may contribute to earlier recognition of HIV infection and improved patient management.

In the future, the integration of routinely available clinical and laboratory parameters into artificial intelligence- and machine learning-based predictive models may enhance the early identification of patients at increased risk of unfavorable outcomes. Further prospective studies involving larger cohorts are needed to validate these findings and to assess their potential role in personalized clinical decision-making.

## 5. Limitations

Several limitations of this study should be acknowledged. First, its retrospective and single-center design may limit the generalizability of the findings to other populations of people living with HIV. In addition, certain biological variables, including CD8+ T-cell counts and neutrophil measurements, were not consistently available in the clinical database throughout the study period, potentially reducing the statistical power of some analyses.

Furthermore, comorbidities, opportunistic infections, and other intercurrent conditions were not systematically evaluated with regard to their potential impact on hematological parameters. The relatively small size of some patient subgroups may have also influenced the statistical significance of certain observed associations.

The study period overlapped with the COVID-19 pandemic, a circumstance that may have affected HIV testing programs, access to healthcare services, and the follow-up of people living with HIV. Moreover, changes in treatment guidelines and variations in the availability of specific antiretroviral regimens during the study period may have contributed to some degree of clinical and therapeutic heterogeneity.

Despite these limitations, the present study provides relevant contemporary data on the hematological profile and prognostic significance of hematological abnormalities among newly diagnosed PLWH from an Eastern European cohort characterized by a high prevalence of late presentation.

## 6. Conclusions

The present cohort highlights the persistent burden of late HIV diagnosis in contemporary clinical practice, with most patients presenting advanced immunosuppression and high viral loads at the time of diagnosis. This biological profile was frequently accompanied by significant hematological abnormalities, reflecting the systemic effects of persistent viral replication, immune dysfunction, and chronic inflammation on hematopoiesis.

Hematological parameters, particularly hemoglobin concentration, lymphocyte count, and platelet count, were significantly associated with immunological and virological markers of disease severity. These findings support the value of complete blood count not only as a potential indicator prompting HIV testing but also as a readily available and informative tool for assessing disease severity at diagnosis. The presence of anemia and multiple cytopenias was associated with unfavorable outcomes and increased mortality during the first year after diagnosis, suggesting that the concomitant involvement of multiple hematopoietic cell lineages may reflect more advanced systemic disease.

Modern antiretroviral therapy was associated with substantial immunological recovery and significant improvement of the hematological profile during the first year following diagnosis, highlighting its effectiveness in achieving viral suppression and restoring hematopoietic homeostasis.

From a clinical perspective, the identification of cytopenias, particularly multilineage cytopenias, should raise suspicion of underlying immunodeficiency and prompt consideration of HIV testing. Furthermore, these abnormalities may contribute to early risk stratification among newly diagnosed patients. The integration of hematological, immunological, and inflammatory markers into prognostic risk models may improve clinical management and support more individualized approaches to the care of people living with HIV.

## Figures and Tables

**Table 1:** Terminology and operational definitions used in the study [13,17,18,19,20,21,22].

Outcome	Definition	Reference
Baseline (BL)	The date of the confirmed HIV diagnosis, established during the patient’s initial medical visit and prior to the initiation of antiretroviral therapy, defined individually for each newly diagnosed case during the study period.	Defined for the purpose of this study
End-point of study (EP)	Defined as the calendar date marking the completion of the first 12 months of clinical follow-up for each individual patient from their respective baseline date.	Defined for the purpose of this study
Mortality	The proportion of patients who died from any cause within the first year after diagnosis relative to the total number of patients included in the study	[17]
Lost to follow-up	Patients with no documented clinical visit within the first year after HIV diagnosis and no evidence of death or transfer to another healthcare facility	[18]
Retention in care	Continued engagement in HIV medical care during the first year after diagnosis, documented by attendance at scheduled clinical visits and/or regular laboratory monitoring.	[19]
Antiretroviral Therapy (ART)	The regular administration of a combination of antiretroviral drugs aimed at suppressing HIV replication	[12]
Acquired Immunodeficiency Syndrome (AIDS) or Advance HIV Disease (AHD)	An advanced stage of HIV infection characterized by a CD4+ T-lymphocyte count below 200/mm^3^ and/or the presence of at least one opportunistic illness specified in Clinical Category C, in accordance with the Centers for Disease Control and Prevention (CDC) immuno-clinical classification systems	[20,21].
Staging of HIV Presentation at Diagnosis		
Early Diagnosis	Baseline CD4+ T-cell count > 350/mm^3^ with no AIDS-defining illness.	[22]
Late Presenter (LP)	Baseline CD4+ T-cell count below 350/mm^3^ and, or presenting with an AIDS-defining illness.	[22]
Advanced HIV Disease (AHD)	Baseline CD4+ T-cell count below 200/mm^3^ or the presentation of an AIDS-defining illness *	[22]
Very Late Presenter (VLP)	Baseline CD4+ T-cell count < 50/mm^3^.	[22]

* This category substantially overlaps with the CDC classification of AHD/AIDS.

**Table 2:** Baseline haematological profile of newly diagnosed HIV patients.

Parameter	Reference Range	Average ± SD	Median (IQR)	Min–Max	Abnormal Values % (*n*)	Notes
Hemoglobin	12–14 g/dL ♀ 14–17 g/dL ♂	12.24 ± 2.41	13	6.2–17.3	34% (35)	2 patients Grade 3 anemia required transfusion
Hematocrit	36–48% ♀ 40–52% ♂	37.92 ± 6.73	38.25	19.8–38.25	32% (35)	
MCV	80–100 fL	82.9 ± 7.58	89.07	62.8–116	12% (13)	9 microcytosis, 4 macrocytosis
Platelets	150–400 ×10^3^/µL	209 ± 76.8	208	30–409	25% (28)	Thrombocytopenia: 21 Grade 1; 6 Grade 2; 1 Grade 3
Lymphocytes	1–4 ×10^3^ /µL	1.788 ± 0.948	1.71	0.18–5.12	21% (23)	20 Lymphopenia 3 Lymphocytosis
CD4	500–1500/µL	262 ± 220	230	6–1074	85% (95)	CD4 Depletion
Neutrophils	2–7 ×10^3^/µL	3.493 ± 2.065	5.9	2.1–9.5	26% (29)	Grade 1 Neutropenia (all)

Legend: WBC: white blood cells; MCV—mean corpuscular volume; grading of cytopaenia—anemia: Grade 1 = Hb 10–11.9 g/dL ♀, Hb 10–13.9 g/dL ♂, Grade 2 = Hb 8–9.9 g/dL, Grade 3 = Hb <8 g/dL; leukopenia: Grade 1 = 3000–3999/µL, Grade 2 = 2000–2999/µL, Grade 3 = <2000/µL; thrombocytopenia: Grade 1 = 100–149 × 10^3^/µL, Grade 2 = 50–99 × 10^3^/µL, Grade 3 = <50 × 10^3^/µL.

**Table 3:** Distribution of combined hematological abnormalities in newly diagnosed PLWH.

Combination of Cytopenias	*n* (%)
Anemia + thrombocytopenia	14 (12.5%)
Anemia + lymphopenia	15 (13.3%)
Anemia + neutropenia	6 (5.3%)
Thrombocytopenia + lymphopenia	12 (10.7%)
Thrombocytopenia + neutropenia	6 (5.3%)
Lymphopenia + neutropenia	11 (9.8%)

**Table 4:** Correlations between immunological, virological, and hematological parameters at HIV diagnosis.

Parameter	CD4 (r)	*p*-Value	HIV RNA (r)	*p*-Value
Hemoglobin	0.374	<0.001	−0.299	0.002
Lymphocytes	0.584	<0.001	−0.349	<0.001
Neutrophils	−0.112	0.436	0.029	0.852
Platelet	0.322	0.0005	−0.222	0.026

**Table 5:** Longitudinal Changes in Hematological and Immunological Parameters from Baseline to 12 Months of Antiretroviral Therapy.

Parameter	Baseline Median	End-Point Median	*p*-Value
CD4 (cells/mm^3^)	244	448	1.1 × 10^−11^
Hemoglobin (g/dL)	13.6	14.9	3.5 × 10^−6^
Platelets (mm^3^)	2.08 × 10^5^	2.37 × 10^5^	0.0005
Neutrophils (mm^3^)	2870	3580	0.201
Lymphocytes (mm^3^)	1810	2090	0.018

**Table 6:** Univariate Analysis of Risk Factors Associated with Mortality during the First Year after HIV Diagnosis.

		Survivor	Death	OR	95% CI	*p*
Age	≥40	26	4	0.76	0.22; 2.57	0.67
	<40	65	13			
Sex	Male	60	13	1.67	0.5; 5.5	0.394
	Female	31	4			
Education	≤8 years	35	8	1.42	0.50; 4.03	0.506
	>8 years	56	9			
LP	Yes	60	13	1.67	0.5; 5.5	0.394
	No	31	4			
VLP	Yes	16	9	5.27	1.76; 15.7	0.0015
	No	75	8			
AHD	Yes	39	15	10	2.15; 46.3	0.0005
	No	52	2			
HbsAg	Yes	17	4	1.33	0.38; 4.62	0.739
	No	74	13			
HCV-Ab	Yes	3	0	0	-	0.447
	No	88	17			
Anaemia <12 g/dL ♀ <14 g/dL ♂	Yes	23	14	13.79	3.63; 52.35	<0.0001
	No	68	3			
Lymphopaenia <1000/mm^3^	Yes	11	9	8.079	2.57; 25.32	<0.0004
	No	79	8			
Trombocytopaenia <150,000/mm^3^	Yes	18	9	4.562	1.54; 13.47	0.0114
	No	73	8			
CRP > 5 mg/L	Yes	38	13	4.19	1.26; 13.88	0.0170
	No	49	4			

Abbreviations: LP, late presenter; VLP, very late presenter; AHD, advanced HIV disease; AIDS, acquired immunodeficiency syndrome; HBsAg, hepatitis B surface antigen; HCV-Ab, hepatitis C virus antibody; OR, odds ratio; 95% CI, 95% confidence interval; CRP, C-reactive protein.

## Data Availability

The original contributions presented in this study are included in the article/Supplementary Material. Further inquiries can be directed to the corresponding author(s).

## Supplementary Materials

The following supporting information can be downloaded at: https://www.mdpi.com/article/10.3390/v18080831/s1, Figure S1: Distribution of baseline plasma HIV-1 RNA levels in the study cohort at the time of HIV diagnosis. Figure S2: Distribution of baseline CD4+ T-cell counts in the study cohort at the time of HIV diagnosis (N = 112). Figure S3: Baseline hematological profile at the time of HIV diagnosis. Figure S4: Distribution of the number of cytopenias at diagnosis and after 12 months of antiretroviral therapy. Table S1: Impact of specific regimen of ART on ARN-HIV suppression and increase of CD4 on end-point. Table S2: Association between systemic inflammation and hematological abnormalities in newly diagnosed PLWH.

## Abbreviations

The following abbreviations are used in this manuscript:

AHD	Advanced HIV Disease
AIDS	Acquired Immunodeficiency Syndrome
ART	Antiretroviral Therapy
BL	Baseline
CDC	Centers for Disease Control and Prevention
CD4	Cluster of Differentiation 4 T Lymphocytes
CI95%	95% Confidence Interval
CNLAS	Romanian National Commission for HIV/AIDS Control
CRP	C-Reactive Protein
EACS	European AIDS Clinical Society
ECDC	European Centre for Disease Prevention and Control
ELISA	Enzyme-Linked Immunosorbent Assay
EP	End-Point of Study
EU-GDPR	European Union General Data Protection Regulation
fL	Femtoliter
g/dL	Grams per Deciliter
HBsAg	Hepatitis B Surface Antigen
HBV	Hepatitis B Virus
HCV	Hepatitis C Virus
HIV	Human Immunodeficiency Virus
IFN-γ	Interferon Gamma
IL	Interleukin
IQR	Interquartile Range
LMNH	Non-Hodgkin Lymphoma
LP	Late Presenter
MCV	Mean Corpuscular Volume
MSM	Men Who Have Sex with Men
OR	Odds Ratio
PLWH	People Living with HIV
RNA	Ribonucleic Acid
SD	Standard Deviation
SK	Kaposi Sarcoma
TARV	Antiretroviral Therapy
TNF-α	Tumor Necrosis Factor Alpha
VLP	Very Late Presenter
W. Blot-HIV	Western Blot HIV
WHO	World Health Organization
µL	Microliter
mm^3^	Cubic Millimeter
