# Supplementary material for: Hematological Profile and Outcomes in Newly Diagnosed Patients with HIV: A 12-Month Single-Centre Cohort Study
Source: Viruses. 2026 Jul 28;18(8):831. doi: 10.3390/v18080831 (PMC13517167; doi:10.3390/v18080831)
Supplement: Supplementary file 1 [file viruses-18-00831-s001.zip › viruses-4405908-supplementary.pdf]

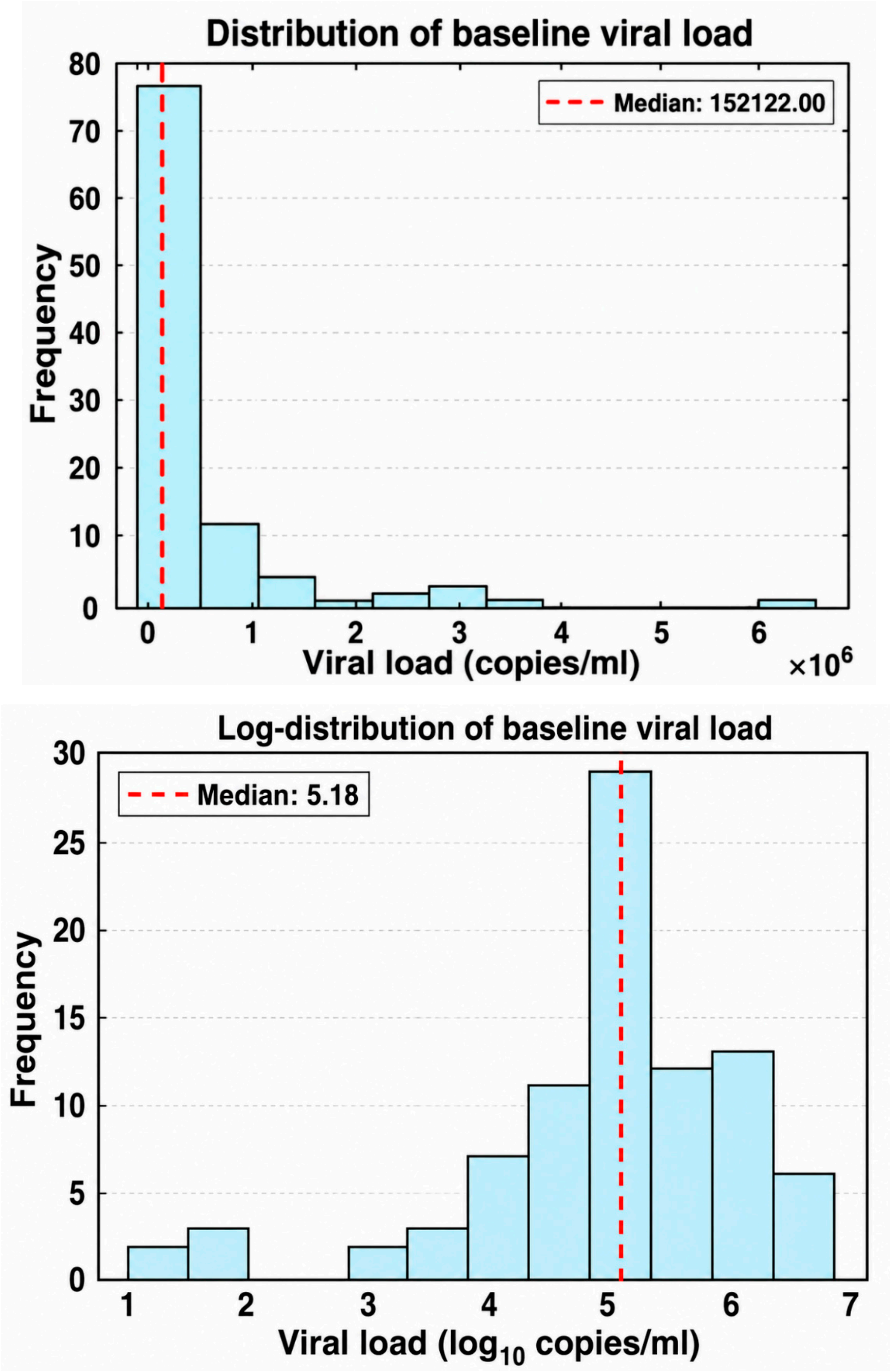

Figure S1: Distribution of baseline plasma HIV-1 RNA levels in the study cohort at the time of HIV diagnosis. The upper panel shows the distribution of raw HIV-1 RNA values (copies/mL), whereas the lower panel presents the log10-transformed distribution to improve visualization of the highly skewed data. The red dashed line indicates the median baseline viral load.

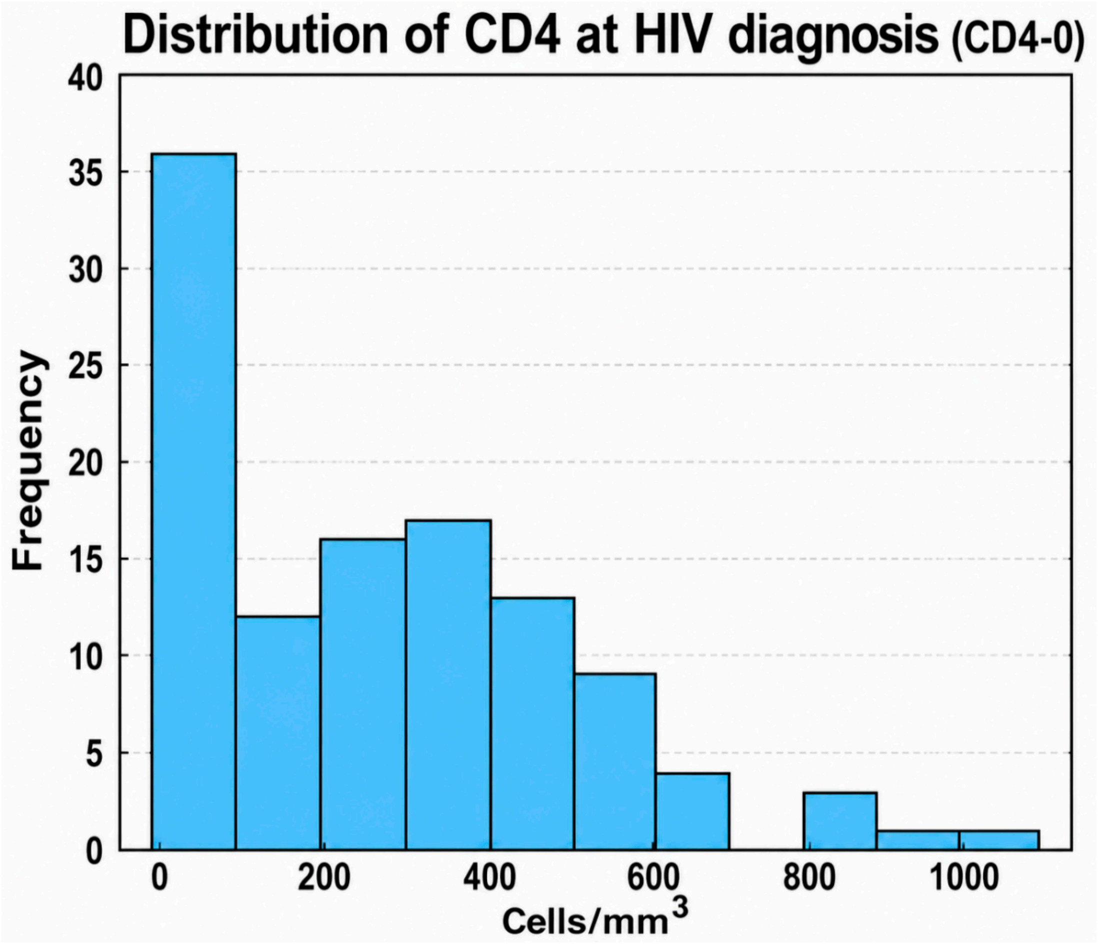

Figure S2. Distribution of baseline CD4+ T-cell counts in the study cohort at the time of HIV diagnosis (N = 112). The histogram illustrates the frequency distribution of absolute CD4+ T-cell counts (cells/mm^3) measured before initiation of antiretroviral therapy. The predominance of lower CD4+ T-cell counts reflects the high proportion of patients presenting with advanced HIV disease at diagnosis.

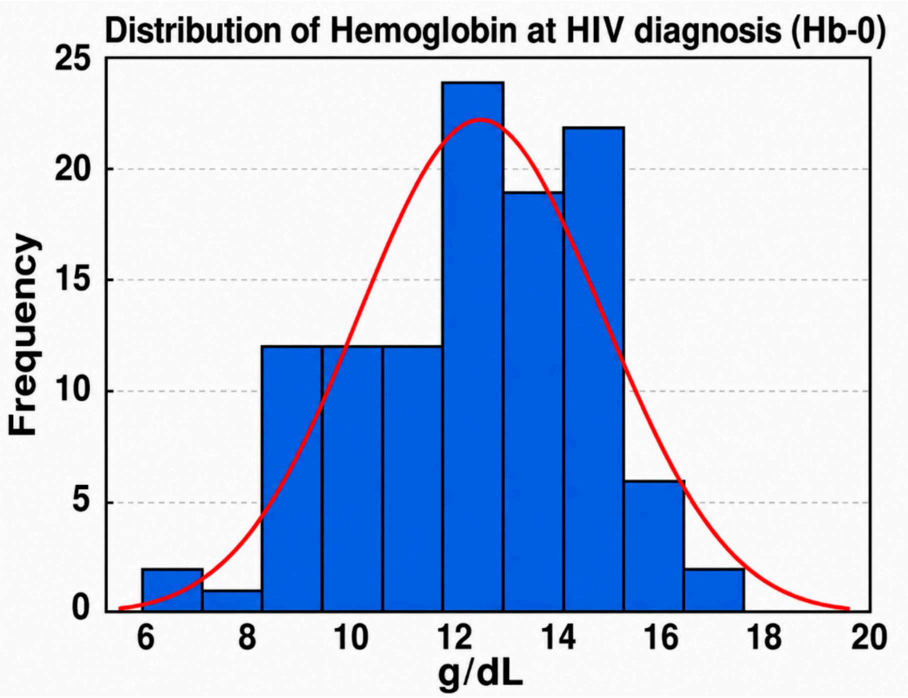

(A) Hemoglobin

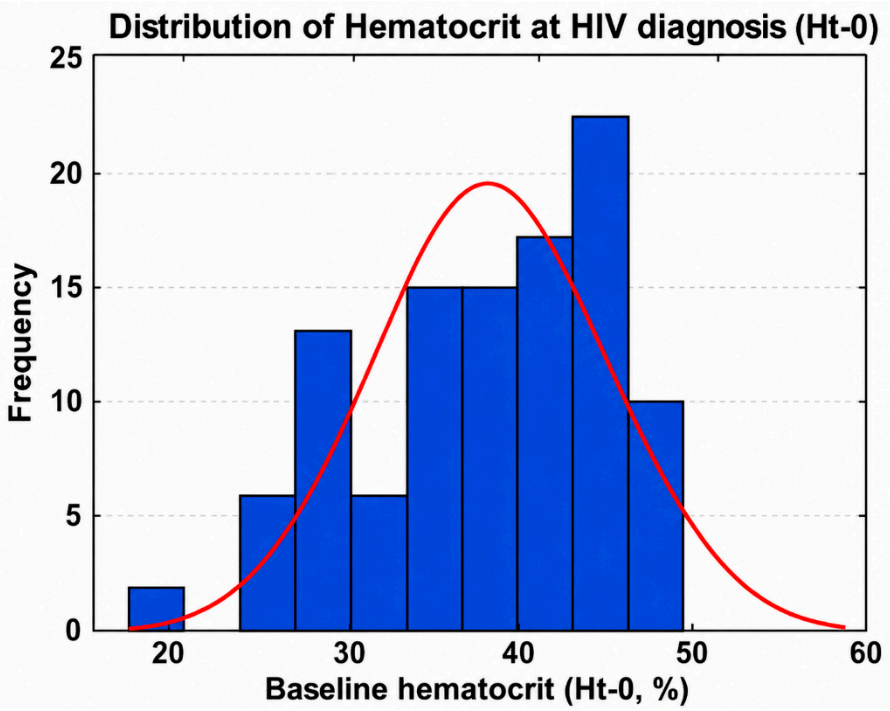

(B) Hematocrit

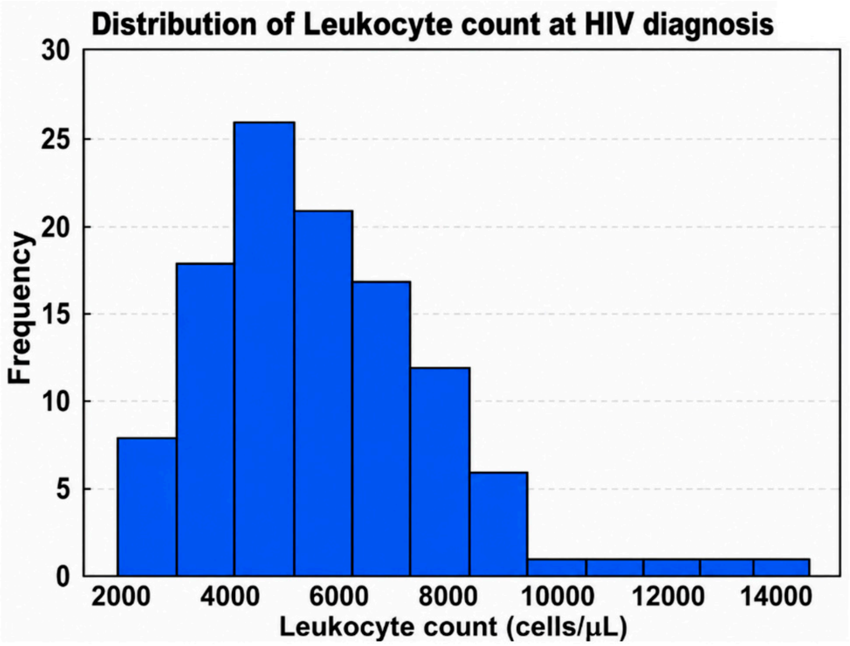

(C) Leukocyte count

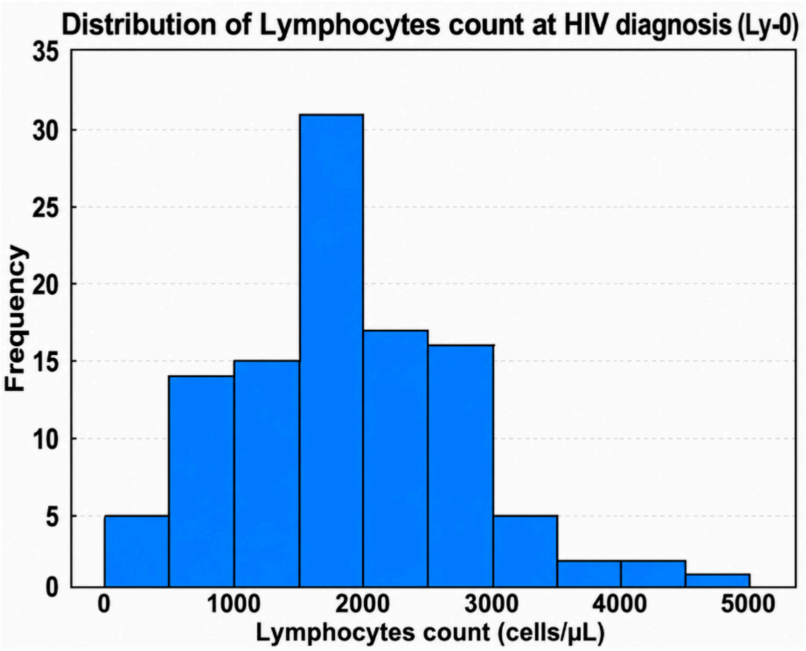

(D) Lymphocyte count

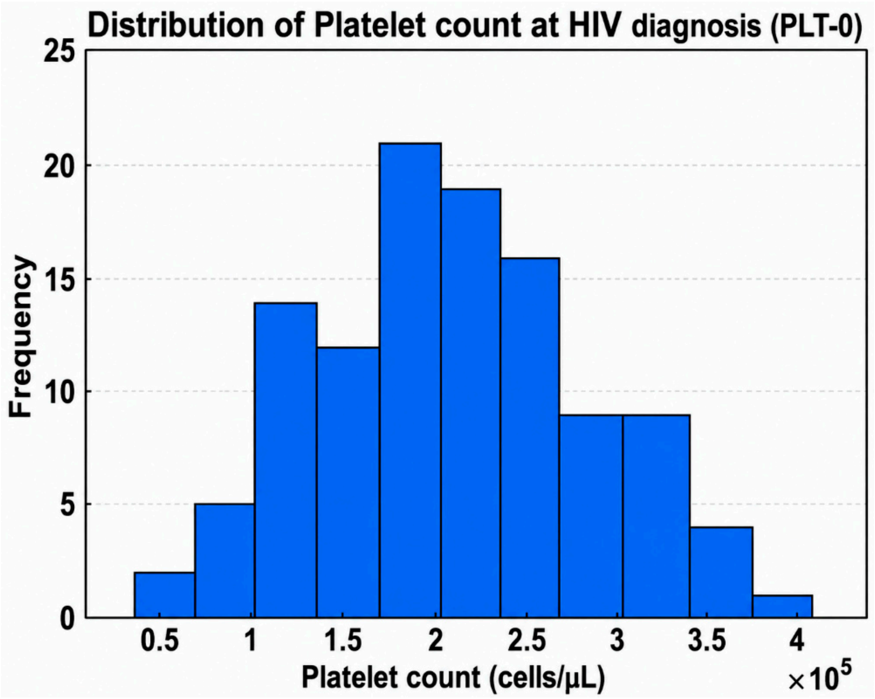

(E) Platelet count

Figure S3. Baseline hematological profile at the time of HIV diagnosis. Histograms illustrate the frequency distribution of (A) Hemoglobin (g/dL), (B) Hematocrit (%), (C) Leukocyte count (cells/µL), (D) Lymphocyte count (cells/µL), and (E) Platelet count (×10³/µL) measured before initiation of antiretroviral therapy. The red curves represent the fitted normal distribution for the corresponding variables.

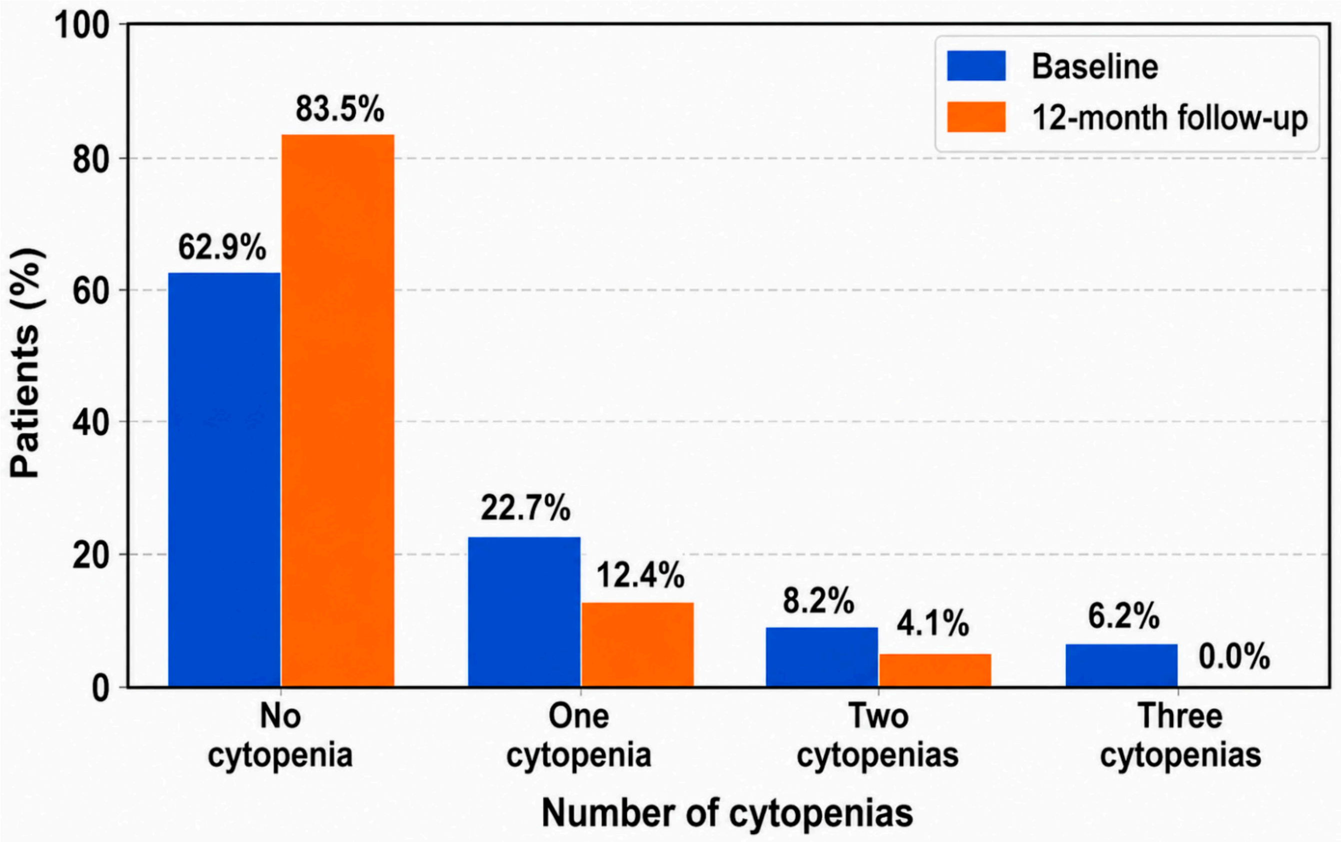

Figure S4. Distribution of the number of cytopenias at diagnosis and after 12 months of antiretroviral therapy

Table S1. Impact of specific regimen of ART on ARN-HIV suppression and increase of CD4 on end-point

Antiviral regimen		N	RNA-HIV (Chi-square Test)					2-Sample t- test	
			UNDET	DET	OR	CI 0.95	p	ΔCD4 Av±SD	p
BIC	Yes	30	25	5	1.12	0.35;3.58	0.845	234±156	0.340
	No	59	49	11				196±209	
GEN	Yes	12	9	3	1.64	0.39;6.82	0.495	151±159	0.241
	No	79	64	13				213±1194	
TRQ	Yes	9	9	64	-	-	0.138	268±206	0.386
	No	80	0	16				198±188	
DOV	Yes	8	7	66	0.628	0.07;5.40	0.672	208.25±139	0.945
	No	81	1	15				204.42±194	
DEL	Yes	7	6	1	0.744	0.08;6.60	0.791	260.85±330	0.647
	No	82	67	15				199.92±175	
ATR	Yes	5	3	2	3.33	0.55;19.91	0.186	31.4±110.21	0.018
	No	84	70	14				215.21±188.92	
RLG+2INRT	Yes	17	13	4	1.53	0.43;5.49	0.507	139.23±195.70	0.134
	No	72	60	12				220.46±186.37	

Legend: 2NRTI, backbone of two nucleoside reverse transcriptase inhibitors; ATR, efavirenz/emtricitabine/tenofovir; BIC, bictegravir/emtricitabine/tenofovir alafenamide; DET, detectable HIV RNA; DEL, doravirine/lamivudine/tenofovir disoproxil; DOV, dolutegravir/lamivudine; GEN, elvitegravir/cobicistat/emtricitabine/tenofovir alafenamide; PI/r, boosted protease inhibitor-based regimen; RLG, raltegravir-based regimen; RPV, rilpivirine-based regimen; TRQ, dolutegravir/abacavir/lamivudine; UNDET, undetectable HIV RNA.

Table S2. Association between systemic inflammation and hematological abnormalities in newly diagnosed PLWH

**Parameter**	**Statistical test**	**Result**	**p value**
CRP vs hemoglobin	Spearman correlation	r = −0.313	0.003
CRP vs leukocyte count	Spearman correlation	r = 0.047	0.664
CRP vs platelet count	Spearman correlation	r = −0.079	0.466
CRP >5 mg/L and presence of cytopenia	Odds ratio	OR = 3.64	0.007

Legend: CRP, C-reactive protein; OR, odds ratio; PLWH, people living with HIV; r, Spearman's correlation coefficient.
